# A Simple fMRI Compatible Robotic Stimulator to Study the Neural Mechanisms of Touch and Pain

**DOI:** 10.1007/s10439-016-1549-y

**Published:** 2016-02-01

**Authors:** F. Riillo, C. Bagnato, A. G. Allievi, A. Takagi, L. Fabrizi, G. Saggio, T. Arichi, E. Burdet

**Affiliations:** Department of Bioengineering, Imperial College of Science, Technology and Medicine, London, UK; Department of Electronic Engineering, University of Tor Vergata, Rome, Italy; Department of Neuroscience, Physiology and Pharmacology, University College London, London, UK; Centre for the Developing Brain, King’s College London, St Thomas’ Hospital, London, UK

**Keywords:** Nociception, Nervous system, Mechatronics

## Abstract

This paper presents a simple device for the investigation of the human somatosensory system with functional magnetic imaging (fMRI). PC-controlled pneumatic actuation is employed to produce innocuous or noxious mechanical stimulation of the skin. Stimulation patterns are synchronized with fMRI and other relevant physiological measurements like electroencephalographic activity and vital physiological parameters. The system allows adjustable regulation of stimulation parameters and provides consistent patterns of stimulation. A validation experiment demonstrates that the system safely and reliably identifies clusters of functional activity in brain regions involved in the processing of pain. This new device is inexpensive, portable, easy-to-assemble and customizable to suit different experimental requirements. It provides robust and consistent somatosensory stimulation, which is of crucial importance to investigating the mechanisms of pain and its strong connection with the sense of touch.

## Introduction

Pain can be intolerable and debilitating. It represents a challenge to scientists, health professionals and to the entire society, given the high costs of preventing or relieving pain and suffering.^11^ While pain is a rapidly advancing field of medicine and biology, many relevant neurophysiological aspects are still unclear. Pain remains difficult to ascertain and is primarily assessed by means of self-report.^24^ The assessment of nociceptive function is crucial in understanding various peripheral neuronal disorders in adults, as well as in studying the early postnatal development of pain perception.

The reliable investigation of the peripheral and central mechanisms of pain requires robust and consistent stimulation patterns. A plethora of commercial and custom devices have been employed to achieve stimulation. Simultaneous excitation of low- and high-threshold mechanoreceptors is often regarded as preferable to other stimulation modalities because of its similarity to natural noxious stimuli,^9^^,^^26^ and is ideal to investigate the ability to discriminate noxious from innocuous contacts. Motor-driven stimulators (reviewed in Baümgartner *et al*.^2^) synchronized to electroencephalography (EEG) acquisition have been developed to deliver mechanical stimuli of different intensities in the non-painful and painful range.

While mechanically evoked potentials guarantee objective assessment of nociceptive functions, the millisecond-range temporal resolution typical of EEG can be combined with the relatively high spatial resolution of functional magnetic resonance imaging (fMRI) in order to gain insights into the mechanisms of pain. Functional imaging techniques have revealed that a distributed set of brain regions are involved in the processing of pain.^22^ fMRI techniques have the potential to advance our understanding of pain at multiple levels (i.e. sensory-discriminatory, cognitive-evaluative and affective-motivational), allowing non-invasive investigation of the neural activity underlying the processing of innocuous and noxious tactile stimulation.

An fMRI compatible system to elicit patterns of tactile stimulation should be able to deliver stimuli of controlled amplitude and frequency which are synchronized with MR image acquisition and other recording equipment. Additionally, fulfilling specific safety and technical specifications and safety standards requires using non-traditional materials and mechatronics.^1^

A few fMRI compatible systems to deliver controlled innocuous and intense tactile mechanical stimulation have been reported in the literature. Kohllöffel *et al*.^14^ proposed to elicit mechanical activation through controlled impacts of a projectile. The projectile consists of a pneumatically accelerated cylinder placed within a guiding barrel, which can be positioned perpendicularly to the targeted skin area. Whilst this device was not originally designed for an MR environment, an adapted system (i.e. containing no metallic parts) was later employed in fMRI experiments by Ringler *et al*.^19^ To investigate the spatial and temporal patterns of cortical activity related to touch and pain perception, Lui *et al*.^15^ used a custom-built pneumatic device with four tips arranged at the corners of a 2 × 2 cm square. Pistons can independently push each of the tips against the skin. Pujol *et al*.^18^ adopted a similar approach, using a specially designed hydraulic system to characterize brain responses to painful pressure in fibromyalgia patients. Dresel *et al*.^8^ presented a pneumatically-driven device employing von Frey filaments^10^ to deliver punctuate tactile stimulation to the subject’s skin.

However, the technical features and parameter range used for stimulation have not been previously described. This type of information is critical to determine the characteristics of the stimulation profiles, knowledge of which is fundamental for correct interpretation of physiological and psychophysical responses.

This paper presents a simple pneumatic system to elicit mechanical innocuous and noxious stimulation of cutaneous receptors, including a detailed description of its properties and performances. In addition to be safe and fully characterized, our system is inexpensive, portable, easy-to-assemble and customizable to suit different experimental requirements. In contrast to previous work, we extensively address the several issues associated with the design of an MR safe and fMRI compatible system, as well as the safety measures required in experiments where high-intensity stimulation can potentially harm the subject. An fMRI experiment demonstrates that the developed system safely and reliably activates brain areas commonly related to pain processing.

## Materials and Methods

A pneumatic system has been developed to produce computer-controlled innocuous and intense mechanical stimulation for investigating associated brain patterns using fMRI. The system consists of: (i) a tactile stimulus interface secured to the stimulation site, and (ii) its control box. Tactile stimulation is produced by a set of projectiles actuated pneumatically. This section describes the system, its characterization and validation.

### Stimulus Interface Design

To consider the requirements of MR safety and fMRI compatibility, and to develop an inexpensive and easy-to-assemble solution, we decided to fabricate our stimulation device using fused deposition of acrylonitrile butadiene styrene (ABS). This material is MR safe, lightweight and has sufficient rigidity.

**Figure 1:**
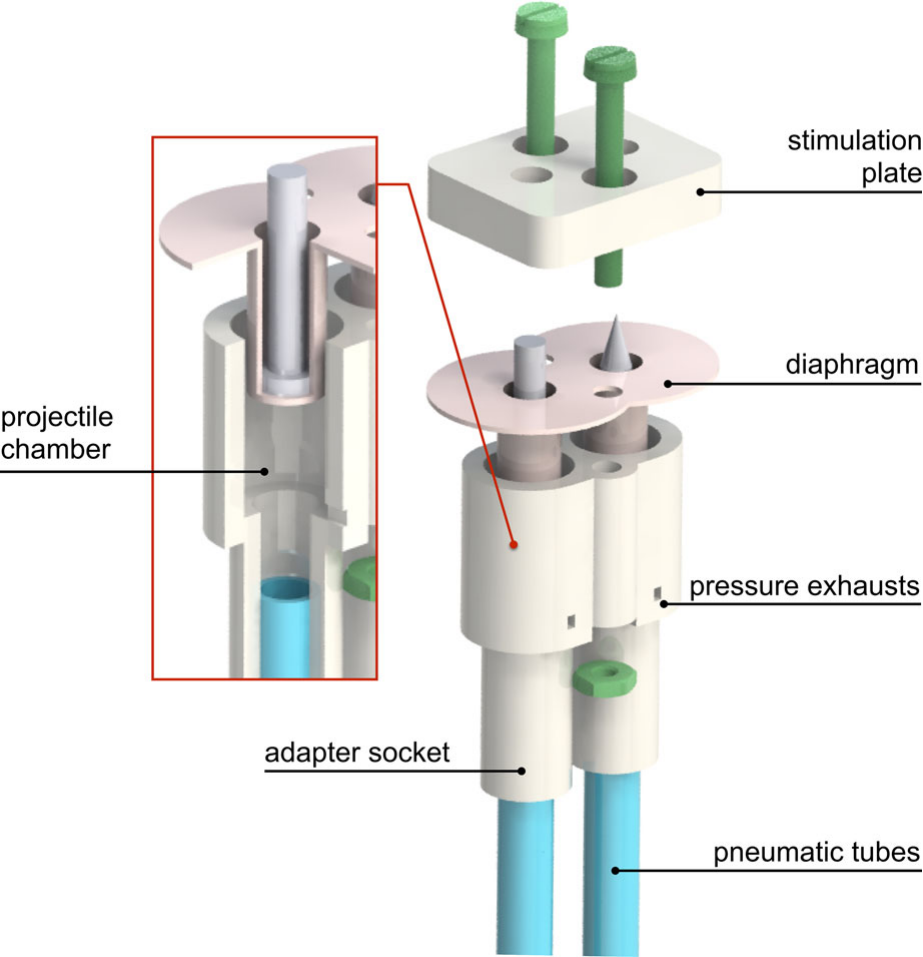
Stimulation interface and section of one projectile chamber. Nylon screws and nuts are used to clamp the diaphragm in between the adapter piece and the stimulation plate; this prevents potential air leakage from reaching the subject’s skin. Both the stimulation frame, here represented in its simplest embodiment, and the projectiles can be designed according to the specific skin area to be stimulated and to the type of stimulation (i.e. intense, innocuous). The projectiles are pushed against the target area when the compressed air fills the projectile chamber and are pulled back at the end of each stimulus as the air is released through the pressure exhausts.

The stimulation device consists of two main rapid prototyped components: a stimulation plate, to be positioned in contact to the skin area to stimulate, and an adapter socket which connects the plate with two projectile chambers fitted with pneumatic tubing couplers (Fig. 1).

The projectiles are driven against the stimulation site through controlled pneumatic actuation. A diaphragm, produced with a two-part silicone moulding kit (of 22 Shore A Durometer scale hardness) and a custom mould, is inserted into the adapter piece and appropriately positioned underneath the projectiles to prevent air from leaking onto the subject’s skin. In addition to preventing the stimuli from generating an undesirable additional tactile sensation (i.e. cooling), this diaphragm is also used to pull the projectiles back to their initial position by exploiting the elastic energy stored during the stimulation phase. Complete retraction of the diaphragm and projectiles is allowed by two openings located at the base of the adapter piece, which act as pressure exhausts.

The design of the frame can be easily adapted to any specific stimulation site and secured to the specific body part with a Velcro^®^ strap. Similarly, the projectiles can be customized to elicit intense rather than innocuous tactile activation at a specific stimulation pressure.

### Controller Architecture

**Figure 2:**
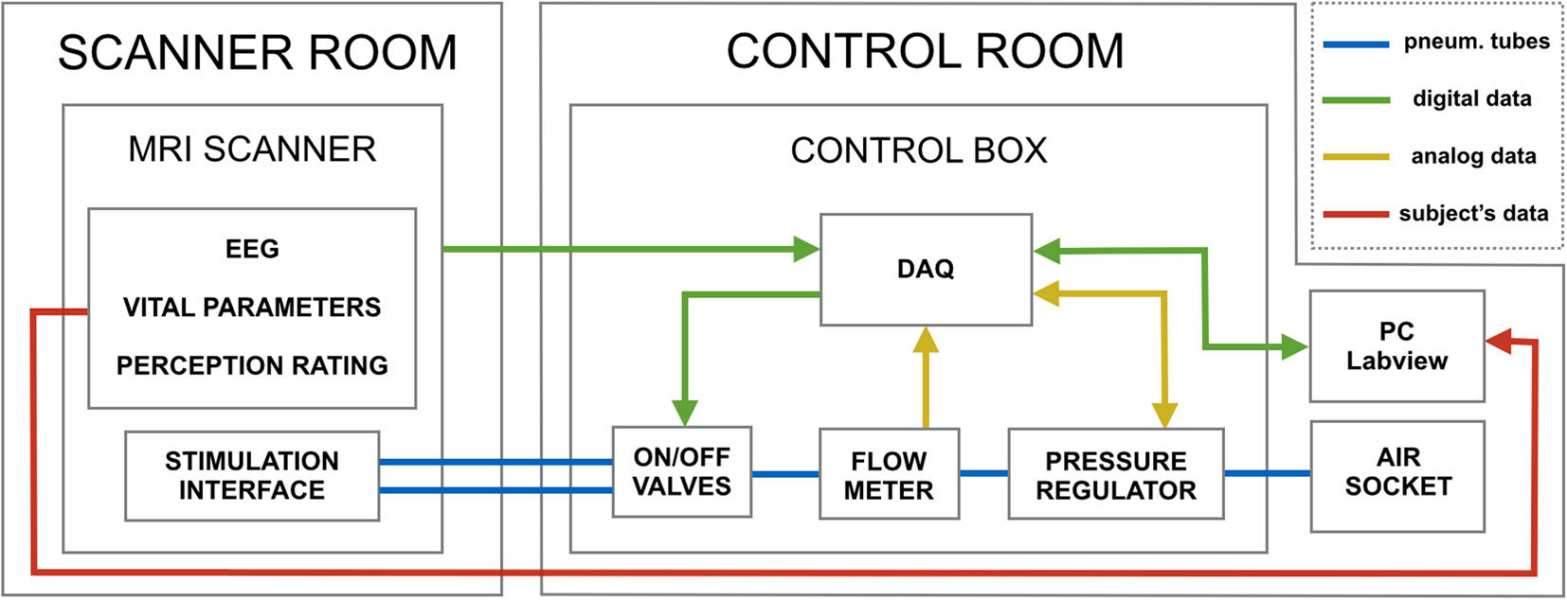
Schematic diagram of our fMRI compatible pneumatic system and its integration within the MR environment. The pressure of the compressed air is modulated by a regulator such that the projectiles included in the stimulation interface are pushed against the subject’s skin according to the values set by the experimenter. The control box also contains a flow meter which allows monitoring force and duration of each stimulus, and on/off valves to control the timing of stimulation. The system is capable of simultaneously acquiring a subject’s fMRI, EEG, vital parameters and perception ratings during each experiment. The experimental protocol is implemented by a dedicated PC through a LabVIEW custom program, which makes use of the MRI scanner’s digital trigger to synchronize the stimulus onset with image acquisitions.

In the control box shown in Fig. 2, a DAQ (Data Acquisition Card) working at a sampling frequency of 1 kHz controls an electro-pneumatic SMC pressure regulator to set and monitor the pressure of the airflow supplied by the hospital compressed breathing air wall socket (or by any external air compressor). The DAQ also controls the duration of the stimuli by operating two ON/OFF electro-mechanical SMC valves, and receives the digital trigger pulses from the MR scanner. The trigger events are used by a custom software (LabVIEW v13; National Instruments, Austin, TX, USA) as a clock to guarantee precise control of the timing of the valve openings and, importantly, to synchronize the stimulation with fMRI image acquisition. A graphical user interface allows the experimenter to set the stimulation parameters and to monitor the measures related to the running experimental protocol.

The subject’s vital parameters (heart rate and oxygen saturations) recorded by an MR compatible Invivo monitoring system (Invivo, Philips Medical systems, Best, NL) and the perception ratings introduced by the subject by means of a custom-built MR safe patient input device are retrieved by the LabVIEW software and stored for later analysis. The software also features the possibility to mark stimulation events, by outputting time stamps to other recording equipment (e.g. electroencephalography system) in correspondence to the occurrence of each stimulus (Fig. 2).

### MR Safety and fMRI Compatibility

An MR safe system does not cause harm to users, subjects and hardware.^12^ Such a system is further fMRI compatible when its materials and workings do not influence the quality of the images, and the functioning of the device itself is not affected by the static magnetic field and electromagnetic pulses of the imaging.^12^

To achieve an inexpensive MR safe and fMRI compatible system requiring simple control, we decided to use an air pressure driven system to generate tactile stimuli with the control box placed outside the MR scanner room. This yields a lightweight, portable and easy-to-assemble experimental apparatus, and guarantees MR safety and fMRI compatibility. Whilst the control box can contain ferromagnetic components as it is positioned in the control room, the tactile stimulus presentation interface needs to be introduced in the electromagnetic field of the MR scanner room and fixed to a subject’s body part. For this reason, our stimulation interface consists of components entirely produced with materials like acrylonitrile butadiene styrene, polyurethane, polymerised siloxanes and Velcro^®^, which are non-conducting, non-metallic, nonmagnetic, and therefore make the device MR safe.

### Redundant Safety Measures

Continuous monitoring is required for experiments where high-intensity stimuli have the potential to injure the subject. In our system, an SMC flow meter is dedicated to constantly acquiring measures during all experimental procedures. The software monitors the flow values and redundantly combines them with the pressure measures provided by the electropneumatic SMC pressure regulator in order to prevent any excessive stimulation force and duration. Mechanical stoppers limit the displacement of the projectiles to avoid their disengagement from the stimulation interface.

In addition to these automatic and intrinsic safety measures, the system features manual mechanisms to halt ongoing stimulation. An emergency red stop button is placed on the top of the control box, so that it is easily visible and reachable by the experimenter; by being pressed, it opens the valves’ supply circuit, immediately interrupting the air flow from the air supply socket to the stimulation interface. Moreover, it is possible to switch off the ON/OFF box button and/or disconnect the +24 V valves’ power supply, both located on the front panel of the control box. The experimenter can also disconnect the control box from the air supply socket.

In order to enable the subject to stop the device, a simultaneous press of the two buttons of an MR safe and compatible custom input device prompts the control program to shut the valves in the control box, thereby immediately interrupting the stimulation. However, should any technical fault prevent the stimulation from being interrupted, the subject can manually detach the pneumatic tubes from the stimulus interface or remove the stimulus interface from the targeted body area. The stimulation interface has been designed such that these two alternatives can be intuitively and effortlessly accomplished.

### Characterization

This paragraph describes the methods adopted to test whether the stimulation force profile is consistent, and determines the delay between the time when a valve is set to open and the subsequent contact of the projectile with the skin. We checked whether the two valves of our apparatus generate identical stimulation profiles, and what is the effect of increasing the air pressure, tube length and valve opening time on physiologically relevant stimulation parameters like duration, force, rise and fall time.

**Figure 3:**
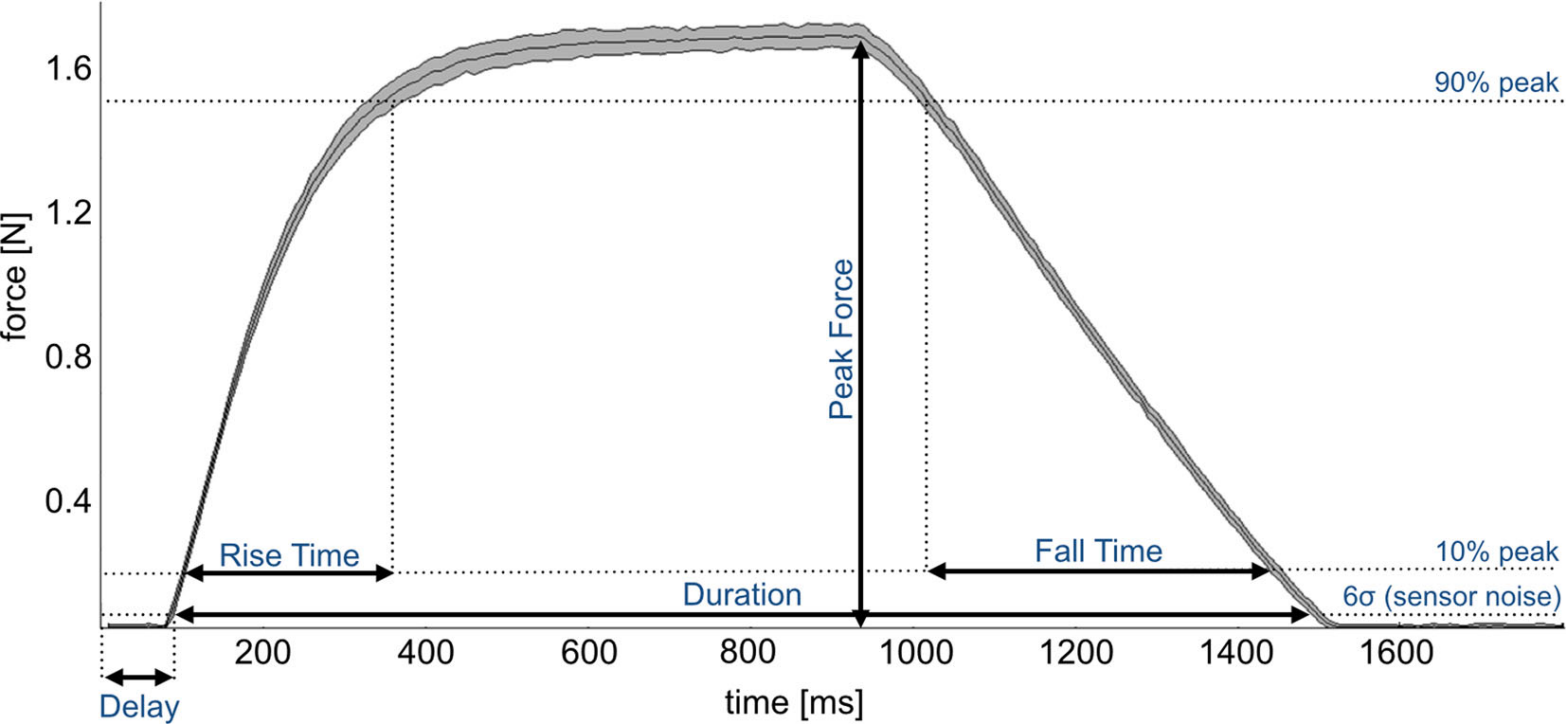
The profile represents the force exerted by a projectile against a strain gauge load cell. Three thresholds (6*standard deviation of load cell rest electrical activity, 10 and 90% peak force) are used to determine delay, duration, peak force, rise time and fall time of our stimuli. The mean and standard deviation represented in this figure have been obtained by averaging 25 force profiles related to a stimulus characterized by the following parameters: tube length = 6 m, valve opening time = 900 ms, pressure = 4 bar.

A Phidgets strain gauge load cell was secured onto the stimulation interface to validate the system and provide quantitative details on the stimulation that it can achieve. The bottom portions of the interface and load cell were firmly clamped together in order to produce reliable measurements, expressing the deformation caused by the impact of the projectile against the top part of the sensor. In this configuration, five measurements were taken for each stimulus (Fig. 3): (i) delay, time interval from the desired stimulus set time and the time when the sensor reading overcomes a threshold (set as six times the standard deviation of the load cell rest electrical activity); (ii) duration, time interval during which the force is greater than the above mentioned threshold value; (iii) peak force of the stimulus force profile; (iv) rise time and (v) fall time, time taken for the contact force to go from 10 to 90% and from 90 to 10% of its maximum value, respectively. Custom software (LabVIEW v13; National Instruments, Austin, TX, USA) was used to trigger the stimuli as well as to read and record the force load cell measurements from a DAQ.

An ample set of conditions were examined, each of them characterized by specific values of air pressure, valve opening time and length of the tube. For every single condition, corresponding stimuli were programmed to be repeated 25 times. In a first test, the length of the tubes was kept constant (at 6 m), and the air pressure was incremented from 2 to 5 bar (in 1 bar steps). For each of the four pressure levels, the valve opening time was changed from 300 to 1200 ms (in 300 ms steps). In a second experiment, the effect of the tube length (from 2 to 8 m, in 2 m increments) was tested at different pressure levels (from 2 to 5 bar, in 1 bar increments), and the valve opening time was kept constant (at 900 ms). Both experiments were carried out twice, the first time connecting the pneumatic tube to the output of one electro-mechanical valve, the second time to the other one, in order to detect any significant difference in the behaviour of the two valves.

### Experimental Validation

Experiments were performed to investigate whether our device can be used to elicit activation in the characteristic cerebral areas associated with innocuous and noxious tactile stimulation. Data from one 28 year old male volunteer with no known medical conditions or recent injury at the level of their fingertips were collected at the Centre for the Developing Brain, King’s College London, St Thomas’ Hospital, London, UK. MR imaging was performed on a Philips Achieva 3-Tesla system (Best, Netherlands). The fMRI data was acquired using a gradient echo echo-planar imaging (GRE-EPI) sequence, with parameters: TR: 1500 ms; TE: 30 ms; FA: 90°; resolution (*x* × *y* × *z*): 3.5 × 3.5 × 5 mm; 22 Slices; SENSE factor: 2. The work was approved by the institutional research ethics committee, and written subject consent was obtained prior to the sessions of data acquisition.

**Figure 4:**
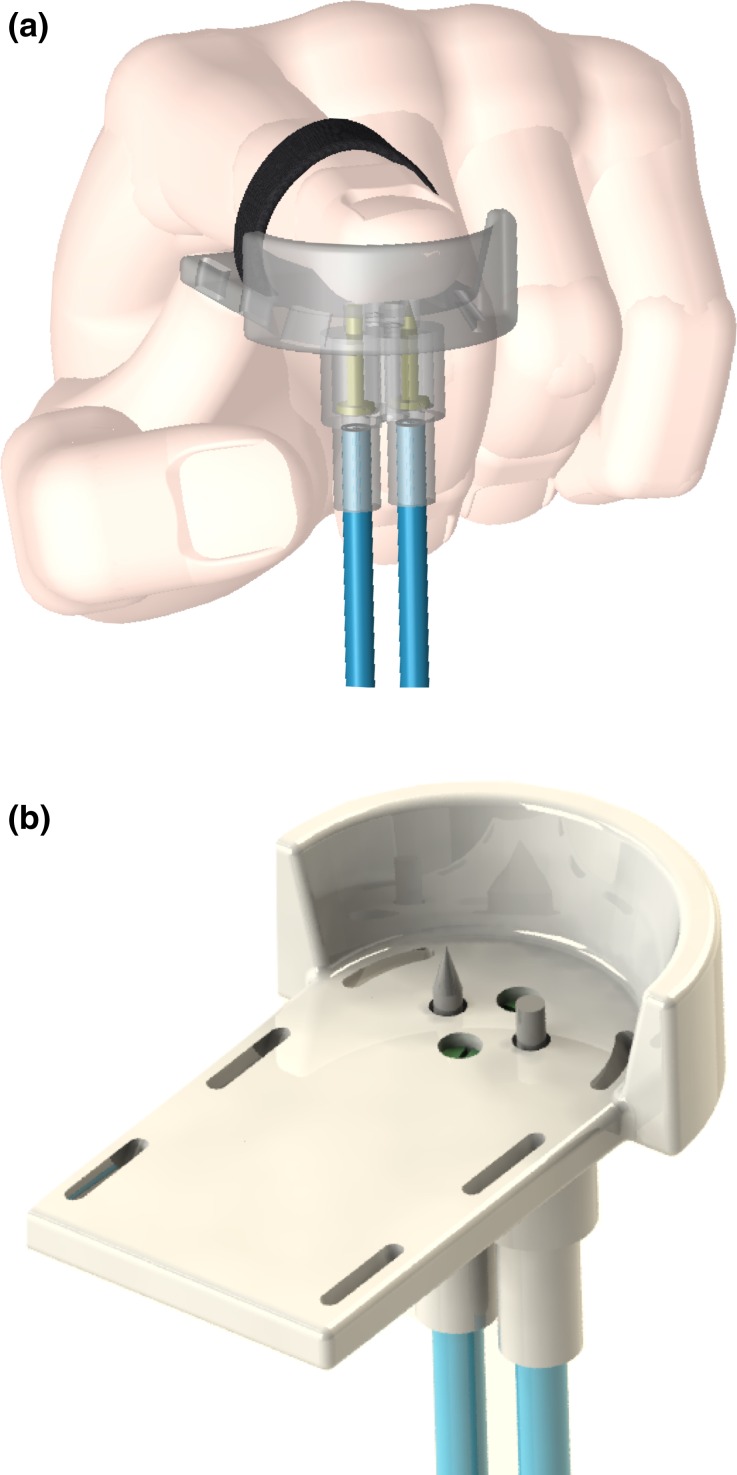
(a) Tactile stimulus presentation interface designed to deliver stimulation at the level of the finger pad (hand CAD courtesy of Joerg Schmit—GrabCAD community). (b) Isometric view of the same tactile stimulus presentation interface. Slots have been included in the design to allow attachment of the subject’s finger to the stimulation frame through Velcro^®^ straps. For illustrations purposes, both projectiles are shown in their active (pushed) configuration.

Innocuous vs. intense tactile stimuli were presented to the subject. An ad hoc stimulation interface was designed to be strapped to their dominant hand index finger (Fig. 4). A psychophysical calibration procedure performed prior to the start of the experiment allowed us to set the air pressure levels in order to present our volunteer with two different degrees of pain (elicited with a sharp projectile), and two equivalent innocuous force intensities (using a blunt projectile). During the calibration session, the experimenter set the air pressure at constant increments of 0.2 bar, starting from a pressure of 1 bar. One stimulus (opening valve time = 900 ms, tube length = 6 m) was presented at each of the set pressure quantities. After each of the stimuli, consisting of the actuation of the sharp projectile against the subject’s index finger pad, the subject was asked (i) whether the stimulus was painful and, if yes, (ii) whether it was severely painful. The levels of pressure corresponding to the first perception of pain (level L) and the one relative to the first appreciation of severe pain (level H) were employed as the two intensities of stimulation to be delivered during the experiment. The same calibration was performed on other 10 subjects (aged 25–30, with no known medical conditions or recent injury at the level of their fingertips) to provide an indicative range of pressure values for which subjects perceive stimulation as painful or severely painful.

The volunteer was then placed into the scanner. Electrodes for electrocardiography were placed to the subject’s chest and a pulse oximetry electrode was applied to one of the fingers. The stimulation paradigm lasted 560 TRs (840 s) and consisted of 5 blocks of 112 TRs (168 s). Within each of the blocks, 4 stimuli were presented, in that both levels of air pressure L and H were used to actuate the sharp and the blunt projectiles against the subject’s fingerpad. The order of the stimuli within each block was randomised before the test. Each stimulus (presented within 1 TR) was followed by 11 TRs (16.5 s) of rest, after which a 100-point visual-analogue scale (VAS) appeared for 6 TRs (9 s) to allow the subject to rate the intensity of the stimulus, using an MR safe and fMRI compatible custom input device. Before a new stimulus was presented, another 10 TRs (15 s) of rest followed the VAS rating. Therefore, stimuli were separated by 40.5 s. This large interval between two stimuli was determined to prevent phenomena of habituation and sensitization.^13^^,^^21^ The choice was also based on preliminary fMRI scans and knowledge of the canonical hemodynamic response to brief stimulation in event-related experimental designs, where a minimum of approximately 30 s is required to ensure adequate return to the baseline signal.^5^

The general linear model (GLM) as implemented in fMRI Expert Analysis Tool (FEAT v5.0.8, part of the FSL image processing package, www.fmrib.ox.ac.uk/fsl) was used to carry out fMRI analysis. Data were preprocessed using standard steps comprising motion correction, slice-timing correction, non-brain tissue removal, spatial smoothing, global intensity normalisation, and high-pass temporal filtering. The design model was specified by convolution of the block design (the binary matrix with 1s representing an event and 0s representing rest) and a set of linear basis functions designed to capture the full range of possible hemodynamic responses induced by stimulation as defined by FLOBS v1.0—FSL’s linear optimal basis sets.^25^

## Results

This section first presents the results obtained from the characterization of our system, showing how delay, duration, peak force, rise time and fall time of the stimulation profiles vary as tube length, air pressure and valve opening time change. Results from the validation test carried out on one healthy male volunteer are then reported.

### Consistent and Adjustable Tactile Stimulation

**Figure 5:**
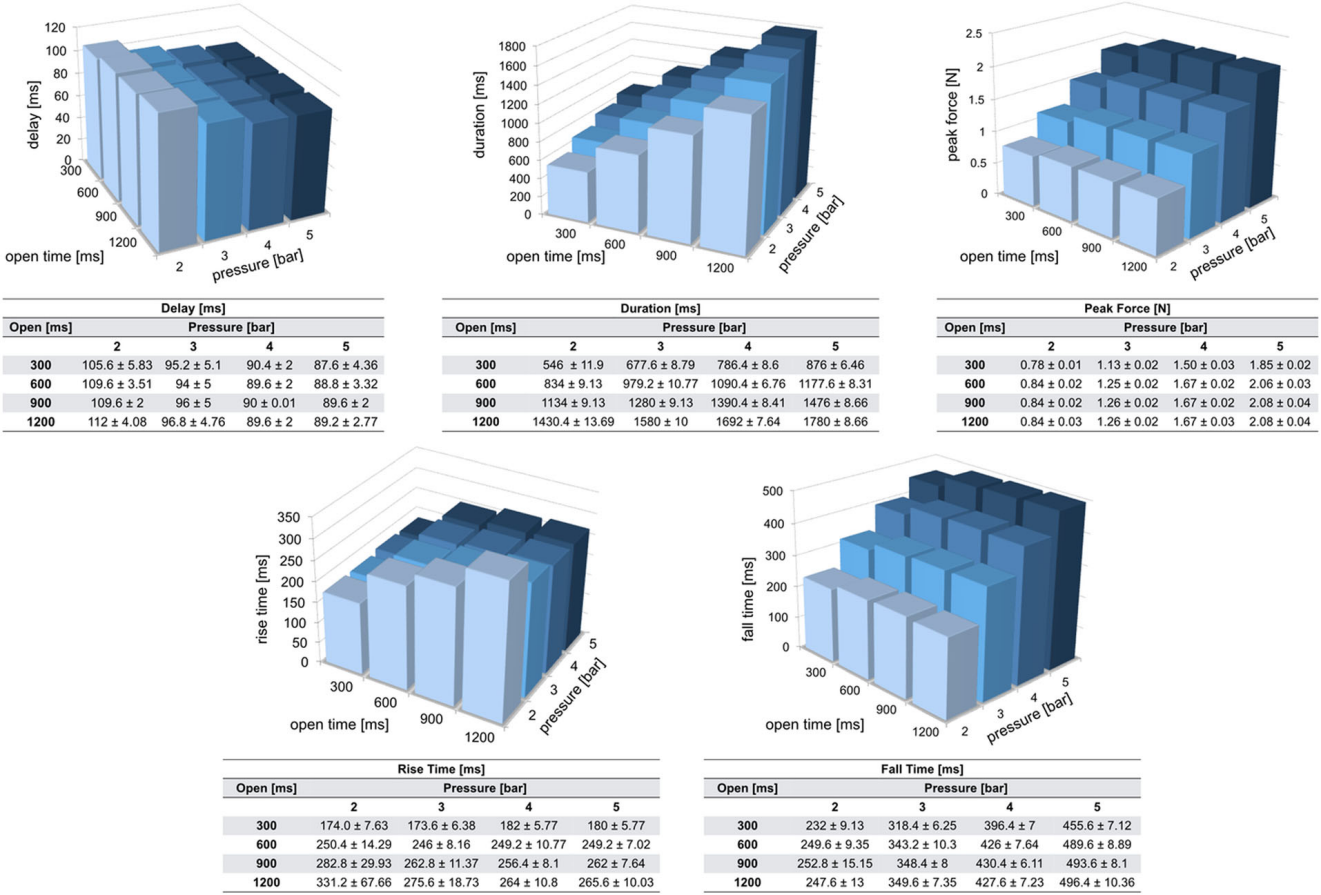
The tables report mean and standard deviation of delay, duration, peak force, rise time and fall time. Mean values are visualised in bar charts. Measurements have been repeated 25 times for each combination of valve opening time and pressure, keeping the tube length fixed at 6 m.

**Figure 6:**
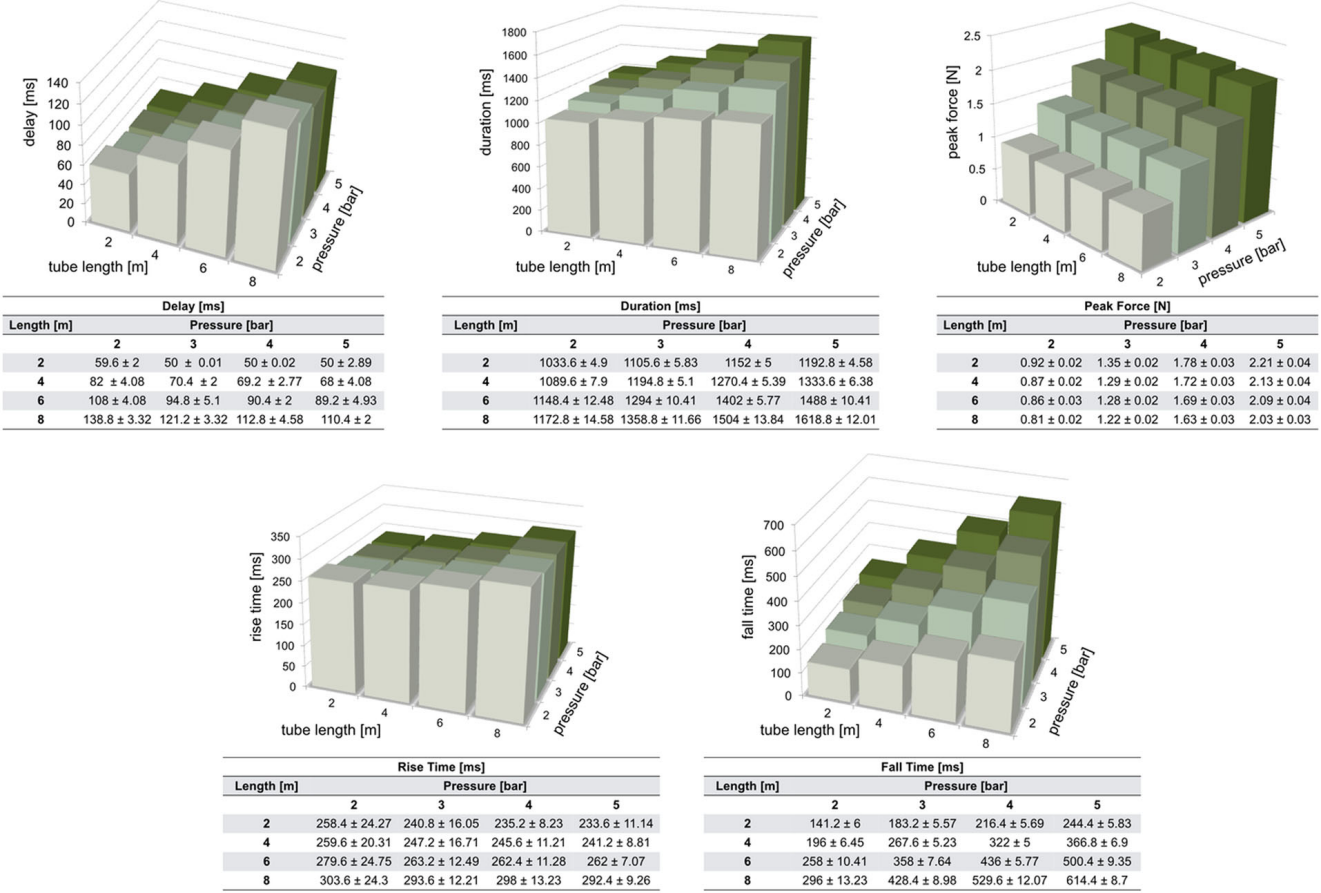
The tables report mean and standard deviation of delay, duration, peak force, rise time and fall time. Mean values are visualised in bar charts. Measurements have been repeated 25 times for each combination of tube length and pressure, keeping the valve opening time fixed at 900 ms.

Figures 5 and 6 show the mean and standard deviation for each of the five types of measurements described in the paragraph Characterization. Visual analysis of the bar charts of Fig. 5 shows the effect of increasing pressure and/or opening valve time on delay, duration, peak force, rise and fall time of the stimulation profiles when the length of the pneumatic tube is kept fixed (6 m). While changing the opening time of the valve does not have a marked effect on the delay, this figure decreases as the pressure increases from 2 to 3 bar, levelling off at higher pressure values. As far as the duration of the stimuli is concerned, both longer valve opening time and greater pressure produce longer stimulation. Considering the peak force reached by the projectiles, higher forces are achieved with greater air pressure. While lower forces are reached with brief stimuli, opening valve times longer than 300 ms do not have an effect on the peak force. An analogous behaviour can be observed for the fall time. The rise time shows comparable values for pressure levels comprised between 3 and 5 bar and opening times between 600 and 1200 ms, while faster increases in force can be obtained by keeping the valve opened for shorter periods (e.g. 300 ms). When setting the air pressure at 2 bar, the rise time increases as the valve opening time becomes longer.

Similar observations can be made for the measures obtained by keeping the valve opening time fixed (Fig. 6). In this case it is possible to study the effect of the tube length on the stimulation profile for each of the pressure values. While longer tubes cause longer delays, duration, rise and fall times, the peak force decreases as the tube length increases. Connecting the pneumatic tube to the output of the other identical electro-mechanical valve leads to results which are consistent to those reported.

Data from the additional psychophysical test conducted on 10 subjects reveal that pain onset at the level of the finger pad was perceived in correspondence of 2.02 ± 0.36 bar and severe pain at 3.42 ± 0.55 bar. Note that these values of pressure to achieve painful responses are specific to the type of projectile, valve opening time and length of the tubes that were set for the test (sharp projectile, opening valve time = 900 ms, tube length = 6 m). Furthermore, the customizability of the stimulation interface enables the experimenter to apply the stimuli over different body parts, each of these characterized by different pain sensitivity.

### Brain Areas Typically Involved in Tactile Processing

**Figure 7:**
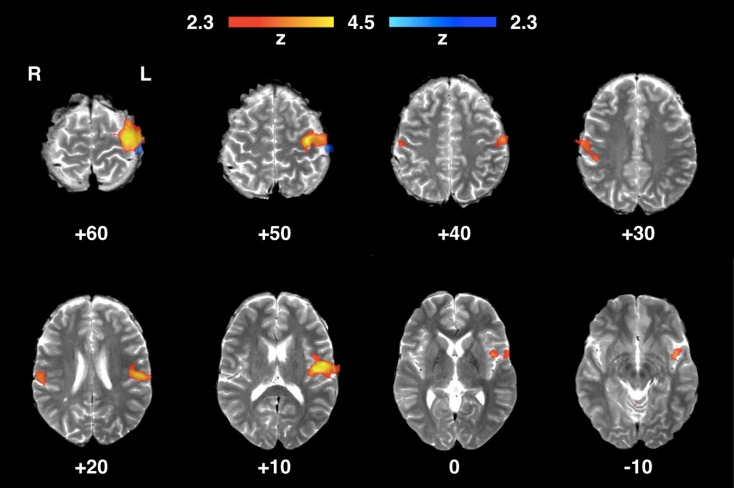
Areas of functional activity identified in the brain of a healthy adult volunteer during innocuous mechanical tactile stimulation of the right hand index’s finger pad. In blue, brain areas activated by lower intensity stimulation (2.6 bar, blunt projectile); in red, brain areas activated by higher intensity stimulation (3.8 bar, blunt projectile).

**Figure 8:**
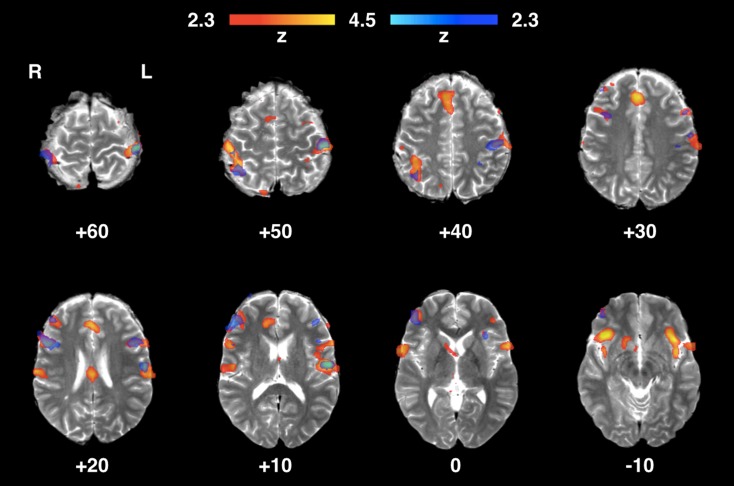
Areas of functional activity identified in the brain of a healthy adult volunteer during intense mechanical tactile stimulation of the right hand index’s finger pad. In blue, brain areas activated by lower intensity stimulation (2.6 bar, sharp projectile); in red, brain areas activated by higher intensity stimulation (3.8 bar, sharp projectile).

The validation experiment identified clusters of functional activity in brain regions characteristically involved in somatosensory and nociceptive processing.^23^ Figure 7 shows the fMRI correlates of innocuous stimulation of the right index finger (blunt projectile) with a well-localised significant cluster of activity primarily in the contralateral (left) primary somatosensory (S1) cortex, but also within the ipsilateral (right) primary somatosensory cortex, and the contralateral secondary somatosensory (S2) cortex. Figure 8 shows the identified patterns of significant brain activity during intense stimulation (sharp projectile) for both intensity levels. Significant clusters of functional activity were identified in the bilateral primary and secondary somatosensory cortices, the supplementary motor area (SMA), the bilateral insular cortices, the anterior cingulate cortex, and the bilateral amygdalae.

Results from the pain intensity ratings reported by the subject after each stimulus through the 100-point VAS confirm that the stimuli delivered by means of the blunt projectile were not perceived as painful (subject did not move the visual-analogue cursor from zero, with zero corresponding to a condition of no pain). Lower intensity mechanical impact by the sharp projectile produced a lower perceptual pain score (49.4 ± 4.9), with respect to the score produced by the higher intensity stimulation (84.8 ± 3.6). Data recorded by the electrocardiography and pulse oximetry electrodes did not reveal changes in heart rate and oximetry following painful stimulation.

## Discussion

We have presented a new MR safe and fMRI compatible system to elicit mechanical innocuous and noxious tactile stimulation, designed to reliably investigate the associated neural mechanisms. The system meets strict safety standards, guaranteed by software and hardware measures implemented to prevent harm to subjects, experimenters and hardware. The choice of pneumatic activation as method to deliver stimulation allows for modularity as well as the control box to be located outside the MR room. Controlled pneumatic actuation generated in the control room is transmitted to the stimulation interface by means of PVC tubes. The stimulation interface is manufactured by means of fused deposition of acrylonitrile butadiene styrene and therefore suitable for experiments in the MR scanner; it also results in a lightweight frame that can be easily secured to the subject’s body part.

While PC-controlled pneumatic MR safe and fMRI compatible systems to elicit mechanical stimulation patterns have been proposed in previous research work, we aimed at developing an easy-to-assemble and inexpensive device, which could also allow customizability and portability to suit different experimental requirements. The equipment is mechanically robust, fits in a small suitcase, can be easily carried, and be installed and removed in less than five minutes. These features are very valuable for fMRI investigations. In an MR environment, a device cannot be permanently installed, and scanner time is usually limited and shared among a large number of users, with priority to clinical work.

In contrast to previous work, we have extensively illustrated its technical features and presented results from the characterization of the system to test the consistency of the stimulation and its reliability. The two electromechanical valves generate consistent stimulation profiles, characterized by corresponding values of delay, duration, peak force, rise and fall time. Our extended characterization allowed for the determination of the stimulation profiles, knowledge of which is essential to meaningfully interpret psychophysical and physiological responses. The characterization also proves that our system is capable of delivering adjustable tactile stimulation; the experimenter can achieve specific measures of duration and peak force for the desired stimulation patterns by regulating parameters such as opening valve time and air pressure. Opening valve times greater than 300 ms do not have an influence on the peak force of the stimulation profiles. This allows experimenters to test the effect of different stimulation duration on brain activation for a specific level of impact force. Although increasing the pressure leads to slight changes in the values of the duration of the stimuli, obtaining control stimuli characterized by same duration and different force can be achieved by adapting the opening time of the valves. The relatively slow rise time is not likely to be of significance for the fMRI experiments for which the system has been designed, given the restrictions on the temporal resolution of fMRI imposed by hardware limitations (usually on the order of a few seconds in a typical GRE-EPI sequence) and due to the measured hemodynamic responses itself (with a peak response usually around 5–7 s). While the temporal limitation of the rise time is expected to prove less suitable for electrophysiological experiments, preliminary tests carried out at the NIHR/Wellcome UCLH Clinical Research Facility on adult healthy volunteers reveal clear EEG activation in response to the patterns of stimulation adopted in the validation experiment presented in this paper.

The forces produced by the system in the tested experimental condition activated brain regions typically involved in pain processing. In accordance with previous relevant literature, we found that the pain generated by the impact of the sharp projectile against the subject’s finger pad was not processed in a single area but in several distributed brain regions.^3^ Significant clusters of functional activation were found in the primary and secondary somatosensory cortices, regions that contribute to our ability to discriminate the location and intensity of painful stimuli.^23^ While the lateral pain system is responsible for the sensory-discriminative pain aspect, the medial pain system is involved in the generation of the affective-motivational dimension of pain.^16^ Within the medial pain system, clusters of activity have been identified in the insular cortex, a structure thought to play a fundamental role in the neural processes underlying the feeling of pain,^7^ and in close proximity to the anterior cingulate cortex, an area reported to be involved in the emotional reaction to pain.^17^ Overlapping clusters of activity following innocuous and intense mechanical stimulation were identified,^15^^,^^20^ with innocuous stimuli evoking spatial patterns of activity consistent with previous studies.^4^

The similarity of intense mechanical tactile stimulation to natural noxious stimuli makes our pneumatic controlled device ideal to shed light on the mechanisms of pain. Not only would this facilitate the definition of objective measures of pain for the population who are unable to report pain, but it would also contribute to the understanding of sensory phenomena like allodynia and hyperalgesia, two conditions characterized by alteration of pain perception. In hyperalgesia, modifications in the central processing cause increased sensitivity to noxious stimuli.^6^ Allodynia is another enigmatic condition for which damage to the peripheral nerve enables recruitment of low-threshold mechanoreceptors,^3^ hence to touch-evoked pain. Our preliminary validation experiment revealed that the system is able to elicit well localised BOLD fMRI response at the level of brain regions typically involved in the processing of mechanical tactile stimulation. In this, the device presented in this work represents an MR safe and fMRI compatible as well as inexpensive solution to achieve controlled and consistent stimulation synchronized with acquisition of relevant physiological measurements.

## Abbreviations

ABS: Acrylonitrile butadiene styrene
BOLD: Blood-oxygen-level dependent
DAQ: Data acquisition card
EEG: Electroencephalography
fMRI: Functional magnetic resonance imaging
GLM: General linear model
GRE-EPI: Gradient echo echo-planar imaging
MR: Magnetic resonance
PVC: Polyvinyl chloride
SMA: Supplementary motor area
TR: Repetition time
VAS: Visual analogue scale
